# Study on the coupling coordination characteristics and influencing factors of ecological environmental civilization and resident public health in China—based on a modified coupling coordination model

**DOI:** 10.1371/journal.pone.0315373

**Published:** 2024-12-06

**Authors:** Qian Xie, Yongkai Wang, Yingchun Zhang

**Affiliations:** 1 Department of Reproductive Medicine, Central Hospital Affiliated to Shandong First Medical University, Jinan, China; 2 School of Statistics and Mathematics, Shandong University of Finance and Economics, Jinan, China; University of Health Sciences Lahore, PAKISTAN

## Abstract

As industrial technologies advance, climate change and environmental pollution increasingly pose threats to human health. This study examines the coupling coordination characteristics between ecological environmental civilization (EEC) and resident public health (RPH) to promote both higher public health standards and enhanced societal sustainability. Utilizing panel data from 31 provinces in China spanning from 2010 to 2022, this paper constructs evaluation indices for EEC and RPH. Initially, the entropy method is employed to determine the development levels of each domain. Subsequently, a modified coupling coordination degree (CCD) model is applied to assess the CCD between EEC and RPH. This research further investigates the spatiotemporal evolution trends of the CCD using methods such as the Dagum Gini coefficient, kernel density estimation (KDE), and Markov chains. Finally, the panel Tobit model is utilized to analyze factors influencing the CCD. Findings reveal that during the study period, both EEC and RPH in China exhibited a stable upward trend, although the overall development level remained relatively low. The CCD showed consistent growth both nationally and across three major regions. Overall inequality in coupling coordination, as measured by Dagum Gini coefficient, has decreased, with the coefficient reducing from 0.0316 in 2010 to 0.0199 in 2022. KDE results indicate a rightward shift in the density curve of the CCD, suggesting a significant reduction in absolute disparities. Panel Tobit regression analysis shows that economic development, urbanization, and education levels significantly and positively influence the CCD on a national scale, with urbanization having the most substantial impact, followed by economic development and education levels.

## 1 Introduction

The United Nations Sustainable Development Goals emphasize the critical need to ensure health and well-being by providing access to universally high-quality healthcare services and by enhancing global health status. However, human activities have significantly altered the global ecosystem, exacerbating various ecological and environmental issues [1,2]. As global environmental challenges intensify, the concept of EEC has become increasingly central to international discourse. This concept not only encompasses ecological balance and sustainable development, but also significantly affects public health outcomes. For instance, the deterioration of ecological conditions, evidenced by increased air and water pollution and biodiversity loss, closely correlates with numerous public health challenges. Specifically, air pollution is directly associated with heightened rates of respiratory and cardiovascular diseases, while water pollution contributes to a rise in waterborne diseases. Furthermore, the diminution of ecosystem services affects both food supplies and the natural purification of air and water, which in turn impacts the quality of human life and health. Additionally, ecological changes indirectly influence RPH by affecting individual lifestyles, socioeconomic statuses, and psychological well-being.

As health issues gain prominence in modern society, it becomes imperative to delve into the intricate relationships between EEC and RPH. An in-depth examination of the interaction mechanisms between EEC and RPH is essential for promoting health and sustainable development objectives. As one of the most populous countries and the second-largest economy in the world, studying China’s environmental and health policies is of significant importance. The rationale for selecting China as the case study for this research includes the following reasons: First, although China has achieved significant improvements in its environmental conditions in recent years, it still faces certain environmental challenges, including air and water pollution, soil degradation, and biodiversity loss, all of which can impact public health. The country’s rapid industrialization and urbanization have exacerbated these issues, making it a critical context for studying the interplay between EEC and RPH. Second, China’s vast geographic area encompasses a wide range of ecological zones and socioeconomic conditions. This diversity allows for a comprehensive analysis of how different environmental and socioeconomic factors influence the coupling and coordination between EEC and RPH across various regions. Third, China maintains extensive datasets on environmental quality, public health, and socioeconomic indicators across its provinces. This availability of detailed and longitudinal data from 2010 to 2022 enables robust empirical analysis and facilitates a nuanced understanding of spatiotemporal trends and influencing factors. Lastly, China has been a pioneer in integrating ecological civilization into its national development strategy. The country’s commitment to ecological modernization, as evidenced by policies like the National Ecological Civilization Pilot Zones and the Beautiful China initiative, provides a rich context for examining the effectiveness of these policies on public health outcomes. Therefore, selecting China as the focus of this study offers a unique and valuable case for investigating the coupling coordination between EEC and RPH. The findings from this research can not only help China advance its sustainable development objectives but also provide guidance for other countries striving to balance ecological sustainability with public health.

Existing studies on EEC and RPH often focus on either ecological environments or public health in isolation, underscoring a significant gap in understanding their interconnectedness and mutual influences.

Research on EEC primarily revolves around the evaluation of ecological environments, performance measurements of ecological civilization, ecological efficiency in specific industries, and the mechanisms influencing EEC. For instance, regarding the evaluation and measurement of ecological environments, Dong et al. (2021) constructed an ecological civilization performance evaluation system and applied Geographically and Temporally Weighted Regression to analyze the determinants of ecological civilization performance in China [3]. Fan et al. (2019) assessed the ecological environments of 31 provincial capitals in China using an evaluation index system characterized by scientific rigor and representativeness [4]. Wu et al. (2021) evaluated the ecological civilization of Chinese provinces for the periods 2012 to 2017 and 2007 to 2012, following China’s first national strategy for ecological civilization construction issued in 2012. Their results indicated that the overall progression rate of China’s ecological civilization from 2012 to 2017 was 14.94%, which is 2.3 times the level recorded between 2007 and 2012 [5]. Jiang et al. (2021) analyzed long-term NDVI data from 1998 to 2018 to assess spatial and temporal trends of ecological environment changes across different regions, provinces, and counties in China [6]. In the case of the Guangdong-Hong Kong-Macao Greater Bay Area, one of the world’s major bay areas, Yang et al. (2020) used comprehensive remote sensing data including vegetation coverage and health indices to propose an integrated ecological quality evaluation index that reveals the spatiotemporal characteristics of ecological quality evolution under urbanization pressure from 1987 to 2017 [7]. Concerning the factors influencing ecological efficiency, Chai et al. (2024) studied the impact of China’s Ecological Civilization Pilot Area policy on ecological efficiency, analyzing mechanisms from the perspectives of environmental penalties, green technological innovation, and environmental propaganda and education [8]. Similarly, Gao et al. (2024) examined the impact of environmental policies on marine ecological efficiency in China’s Ecological Civilization Pilot Areas, discussing the policy’s mechanisms from national and regional perspectives [9]. Li et al. (2020) constructed an evaluation system that includes indicators such as the greening coverage rate of built-up areas to assess the ecological environment of 19 resource-based cities in Northeast China, examining their coordinated development with the socio-economic system using a coupling coordination model [10]. Gu et al. (2020) discussed the integration of ecological civilization into China’s national strategy for sustainable development and how governmental administrative system reforms embed environmental protection in political, economic, and social systems. Furthermore, they provided policy recommendations to strengthen the implementation of ecological civilization to contribute to global sustainable development [11].

On the public health front, extensive scientific research has been conducted by clinicians, public health scholars, and interdisciplinary researchers. Representative studies largely rely on survey data to scientifically evaluate RPH. For instance, Li et al. (2023) utilized data from the China Family Panel Studies (CFPS) to explore the effects of environmental pollution and renewable energy on residents’ physical and mental health, finding that the development of renewable energy could partially offset the negative health impacts of environmental pollution [12]. Girard and Nocca (2020) highlighted pollution as a significant factor affecting human health and well-being [13]. Liu et al. (2023) used the 2017 China General Social Survey data and a hierarchical linear model to scientifically analyze the impact of air pollution on public health, discovering that air pollution significantly negatively affects resident health [14]. Currie et al. (2009) combined data from birth certificates detailing current residences with air quality monitoring information to examine the impact of three common air pollutants on infant health in New Jersey during the 1990s. Their findings consistently showed the negative impact of carbon monoxide exposure on infant health, both prenatally and postnatally [15]. Zhu and Lu (2023) used data from the China General Social Survey to measure self-rated health, mental health, and perceptions of air pollution, alongside socioeconomic indicators and objective air pollution data. Their study found that perceived air pollution significantly negatively impacts self-rated health and mental health, while objective air pollution significantly adversely affects mental health but not self-rated health significantly [16]. He et al. (2016) utilized a time-series analysis incorporating data on air pollution, weather conditions, and non-accidental fatalities from Ningbo, China, covering the period from 2009 to 2013. This analysis distinctly quantified the impact of air pollution on years of life lost (YLL), emphasizing the critical and immediate need for controlling air pollution [17]. Guo et al. (2022) employed the Probit model with panel data from the CFPS to examine the impact of income disparities in rural areas on public health [18]. Pei et al. (2023) used data from the China General Social Survey to empirically analyze the impact and mechanisms of environmental regulation on public health, finding that environmental regulations significantly improve health levels, with heterogeneity across different populations [19]. Yang et al. (2020) analyzed data from the "2010 Third Survey on the Status of Women in China," studying disparities in environmental awareness between urban and rural residents. They found that urban residents had a higher awareness of air pollution, water pollution, garbage pollution, and noise pollution, and that perceived air and noise pollution adversely affected their health [20]. Xie et al. (2024) matched panel data from the 2018 CFPS with the national forest city construction list and employed Probit, Logit, and instrumental variables methods to explore the relationship, varied effects, and underlying mechanisms linking national forest cities to resident health. Their results demonstrated that the development of national forest cities markedly enhances both the physical and mental health of residents, with these conclusions holding steady across multiple robustness checks [21].

The preceding literature review reveals a comprehensive examination of EEC and RPH. However, as two interdependent systems, EEC and RPH mutually influence and constrain each other. Current research primarily focuses on the unidirectional impact of EEC on RPH [22–24], with relatively few studies addressing their coupled and coordinated relationship. This study is structured around two key scientific questions: (1) How has the relationship between EEC and RPH in China evolved? (2) What are the main factors influencing their coordinated development?

To address these questions, this paper establishes an evaluation index system for both EEC and RPH. By scientifically measuring the development levels of these two systems, a modified CCD model is used to analyze their interrelationship in China from 2010 to 2022. Additionally, this study analyzes the spatiotemporal trends of their coupling coordination and employs a panel Tobit model to explore external influencing factors.

This study makes several significant contributions to the existing body of knowledge on the interaction between EEC and RPH, particularly within the context of China. These contributions can be summarized as follows: First, while previous studies have largely examined EEC and RPH independently, this research bridges the gap by investigating their coupled and coordinated relationship. By constructing comprehensive evaluation indices for both EEC and RPH, the study provides a holistic framework to understand how these two domains interact and influence each other. Second, the study introduces a modified CCD model to accurately assess the coupling coordination characteristics between EEC and RPH. This methodological innovation allows for a more nuanced analysis of the interrelationship between ecological environment and public health, which has not been extensively explored in prior research. Third, utilizing panel data from 31 provinces in China from 2010 to 2022, the study offers a detailed examination of the spatiotemporal trends in the coupling coordination of EEC and RPH. Methods such as the Dagum Gini coefficient, KDE, and Markov chains are employed to analyze these trends, providing insights into the regional disparities and temporal evolution of the CCD. Fourth, the research employs a panel Tobit model to identify and analyze the external factors influencing the CCD. The findings highlight the significant roles of economic development, urbanization, and education levels in enhancing the coordination between EEC and RPH. This aspect of the study contributes to the understanding of how socio-economic variables impact the synergy between environmental sustainability and public health.

**Fig 1:**
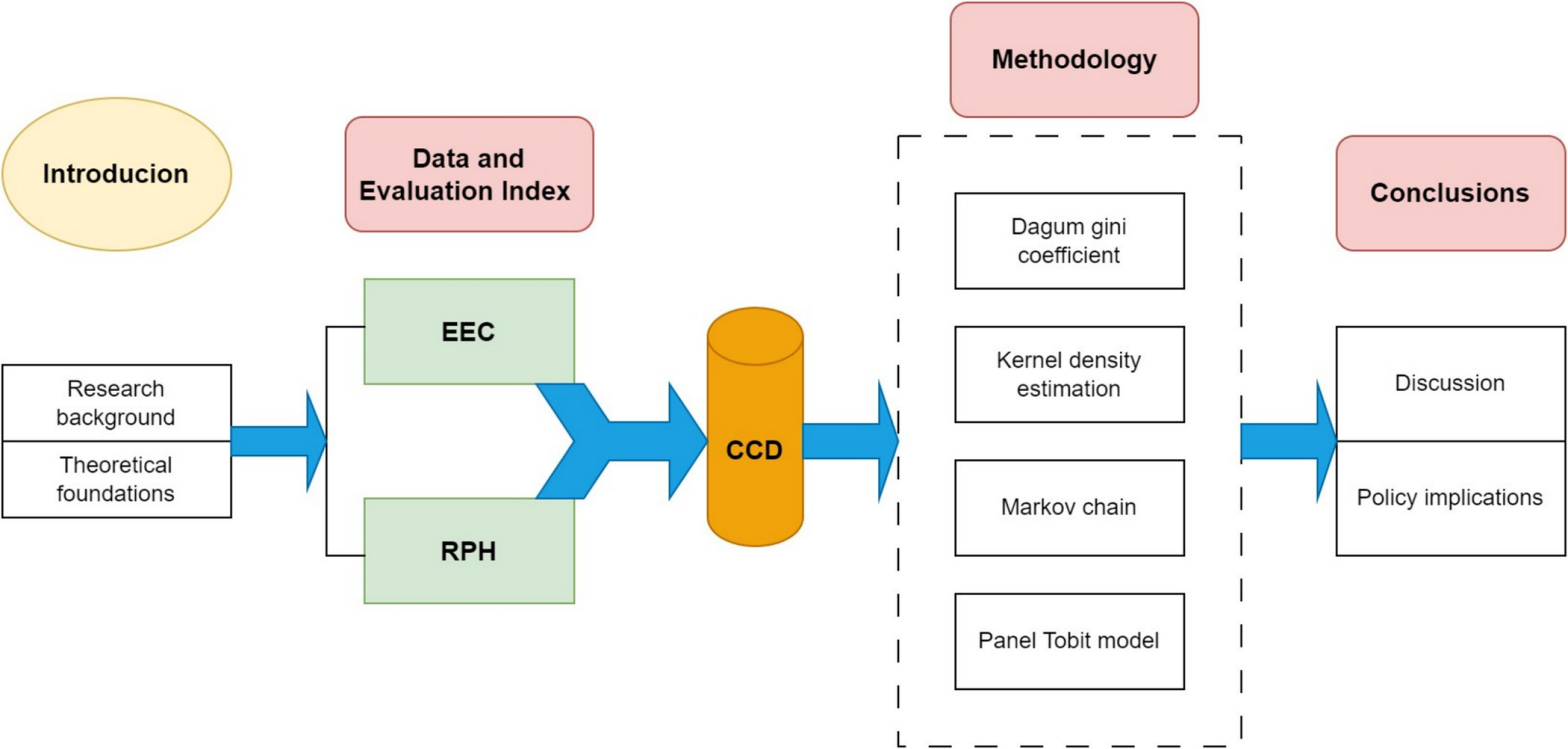
The framework of this paper.

In summary, this paper advances the understanding of the complex interplay between EEC and RPH by integrating their assessment, developing a modified analytical model, examining spatiotemporal trends, identifying key influencing factors, and offering actionable policy recommendations. These contributions collectively address a critical gap in the existing research and provide a foundation for future studies in this interdisciplinary field. The structure of the article is illustrated in Fig 1.

## 2 Evaluation index system and methodology

### 2.1 Evaluation index system

In assessing EEC and RPH, the academic community has not yet reached a consensus. Scholars have developed various evaluation indices based on their research objectives; however, these systems often lack uniformity.

Regarding the evaluation indicators for EEC, there are primarily two methods. The first method uses single indicators. For example, Dong et al. (2021) constructed a performance evaluation system for ecological civilization and employed Data Envelopment Analysis to measure the ecological civilization efficiency of 30 provinces and cities in China [25]. Some scholars have also used quasi-natural experiments, such as China’s national ecological civilization pilot zones [26] and national forest city pilot policies [27], as proxy variables for empirical studies on ecological civilization. The second method involves the construction of comprehensive indicator systems. Zhang et al. (2020) determined indicators from five dimensions—ecological economy, ecological society, ecological culture, ecological environment, and ecosystems—to evaluate China’s ecological civilization construction [28]. Mi et al. (2022) built an ecological civilization index system that includes 28 specific indexes, considering the representativeness of indicators and data availability, based on the conception and overall direction of ecological civilization [29]. Zhang et al. (2019) developed an ecological civilization index system at the national level that includes green production, green environment, green infrastructure, and green living [30]. Meng et al. (2021) constructed an evaluation indicator system for urban sustainable development, focusing on green sustainability, green development, and green coordination at the city level [31].

Currently, the measurement and quantification of RPH mostly rely on single indicators, such as morbidity and mortality rates. However, some scholars have attempted to construct more comprehensive evaluation systems. For instance, Jia et al. (2023), considering the availability of relevant data, selected six variables including perinatal mortality to build an evaluation system for residents’ physiological health [32]. Yang et al. (2022) designed a self-assessment scale that includes mental health, physical health, and well-being to quantitatively analyze the general health levels of residents [33]. Nevertheless, there remains a relative scarcity of research on methods for measuring public health.

This paper, drawing on documents such as the "Evaluation System for Ecological Civilization Construction" issued by China’s National Development and Reform Commission and the "National Basic Public Health Service Standards (Third Edition)" issued by the National Health and Family Planning Commission, builds on existing research [34–38] to construct evaluation index systems for both EEC and RPH. These systems consist of criterion layers and element layers. The EEC index system includes three primary indicators: EEC pressure, EEC status, and EEC response. The RPH index system comprises four primary indicators: medical staff, medical facilities, healing capacity, and medical efficiency. The weights of each indicator are determined based on the information entropy. The specific evaluation index systems for both domains are detailed in Table 1.

**Table 1:** Evaluation index system of EEC and RPH.

System	Target layer	Indicator layer	Unit	Attribute	Weight
EEC	EEC pressure	Chemical oxygen demand emissions	Tons/10^8^ yuan	-	0.0137
		Industrial sulfur dioxide emissions	Tons/10^8^ yuan	-	0.0258
		General industrial solid waste emissions	Tons/10^4^ yuan	-	0.0218
	EEC state	Per capita water resources	cubic meters	+	0.4921
		Per capita park and green space area	square meters	+	0.1101
		Green coverage rate in built-up areas	%	+	0.0251
	EEC response	Harmless treatment rate of household waste	%	+	0.0514
		Urban sewage treatment rate	%	+	0.0250
		Green finance	Score	+	0.2351
RPH	Medical staff	Number of practicing (assistant) physicians per 10,000 people	persons	+	0.0904
		Number of registered nurses per 10,000 people	persons	+	0.0758
		Number of public health managers per 10,000 People	persons	+	0.1231
		Number of primary health care workers per 10,000 People	persons	+	0.0614
	Medical Facilities	Per capita total assets of health institutions	Yuan	+	0.1055
		Number of hospital beds per 1,000 people	Number	+	0.0639
		Number of hospitals per 10,000 people	Number	+	0.1030
		Number of primary health care institutions per 10,000 people	Number	+	0.0806
	Healing Capacity	Maternal mortality rate	1/10^5^	-	0.0081
		Perinatal mortality rate	‰	-	0.0128
		Number of tertiary hospitals	Number	+	0.0976
	Medical Efficiency	Daily average number of patients treated by a physician	Number	+	0.0934
		Daily average number of hospital bed-days managed by a physician	Days	+	0.0458
		Hospital bed utilization rate	%	+	0.0303
		Average hospitalization days for patients	Days	+	0.0085

### 2.2 Data sources

Due to data availability issues, this study has selected the 31 provinces of China, spanning from 2010 to 2022, as research samples, excluding Hong Kong, Macau, and Taiwan. Data on EEC indicators are sourced from the China Social Statistics Yearbook, the China Research Data Services Platform (CNRDS), and the digital inclusive finance index by Peking University [39]. Data on RPH indicators are derived from the China Health Statistics Yearbook, the China Health and Wellness Statistics Yearbook, the China Health and Family Planning Statistics Yearbook, and the China Economic and Social Big Data Research Platform. For missing data in certain years or provinces, interpolation methods were applied for imputation.

### 2.3 Research methods

#### 2.3.1 Entropy method

The entropy method is a commonly used objective weighting method that has the advantage of determining the weights of indicators without the influence of subjective factors. Consequently, this paper employs the entropy method to measure the levels of EEC and RPH. The model construction process begins with the standardization of data to eliminate differences in dimensions. This is followed by determining the weights through the information entropy of each indicator. The specific process is as follows:

1. Dimensionless normalization of indicators.
$$Positive\;indicators:\;x_{ijt}^{'}=\frac{x_{ijt}-\min\{x_{ijt}\}}{\max\{x_{ijt}\}-\min\{x_{ijt}\}}$$ (1)
$$Negative\;indicators:\;x_{ijt}^{'}=\frac{\max\{x_{ijt}\}-x_{ijt}}{\max\{x_{ijt}\}-\min\{x_{ijt}\}}$$ (2)
2. Calculation of indicator proportions and information entropy.
$$Indicator\;proportion:\;p_{ijt}=\frac{x_{ijt}^{'}}{\sum_{i=1}^{n}\sum_{i=1}^{m}x_{ijt}^{'}}$$ (3)
$$Information\;entropy:\;e_{j}=-\frac{1}{\ln\;m}\sum_{i=1}^{n}\sum_{t=1}^{m}\left(p_{ijt}\times \ln\;p_{ijt}\right)$$ (4)
3. Calculation of indicator weights.
$$w_{j}=\frac{1-e_{j}}{\sum_{i=1}^{n}\left(1-e_{j}\right)}$$ (5)
4. Calculation of the system score.
$$U_{i}=\sum_{j=1}^{n}w_{j}x_{ijt}^{'}$$ (6)

In the formulas above, *x*_*ijt*_ represents the value of indicator *j* for province *i* in *t* year; *e*_*j*_ is the entropy value of indicator *j*; *w*_*j*_ is the weight of indicator *j*; and *U*_*i*_ are the comprehensive scores of the two systems, respectively.

#### 2.3.2 Modified coupling coordination model

The CCD model is an analytical method used to assess the interaction and coordinated development between two or more systems. It is commonly employed in fields such as environmental science and management science to evaluate the coordination and interdependence between different systems or subsystems. Jiang et al. (2017) identified issues with value errors in the traditional CCD model, which could not achieve optimal reliability and validity [40]. Drawing on research by Wang et al. (2021) [41], this paper utilizes a revised CCD model to examine the coupling and development levels between EEC and RPH systems.

In the traditional CCD model, the CCD values do not uniformly distribute over the [0, 1] interval, leading to misinterpretations of coupling relationships. Additionally, when subsystems are assumed to have equal importance, the traditional model’s coordination degree *D* can be overly simplified, reducing its effectiveness in representing true coordination levels. The revised CCD model directly addresses these issues by modifying the traditional CCD model. Since the traditional coupling degree *C* is not evenly distributed over the [0, 1] interval, the validity issues stem from the model itself. The modifications to the model are as follows:

$$C=\sqrt{[1-\frac{\sum_{i>j,j=1}^{n}\sqrt{{\left(U_{i}-U_{j}\right)}^{2}}}{\sum_{m=1}^{n-1}m}]\times {\left(\prod_{i=1}^{n}\frac{U_{i}}{\max U_{i}}\right)}^{\frac{1}{n-1}}}$$ (7)

$$T=\sum_{i=1}^{n}\alpha_{i}\times U_{i},\;\sum_{i=1}^{n}\alpha_{i}=1$$ (8)

$$D=\sqrt{C\times T}$$ (9)

Here, *U*_*i*_∈[0,1], *C*∈[0,1], the more dispersed the subsystems are, the lower the *C* value; conversely, the closer the subsystems are, the higher the *C* value.

In this study, since there are only two subsystems, EEC and RPH, *n* = 2. *U*_2_ defined as max *U*_*i*_, assuming *α*_1_ = *α*_2_ = 0.5,the specific model setup in this paper is as follows:

$$C=\sqrt{[1-(U_{2}-U_{1})]\times \frac{U_{1}}{U_{2}}}$$ (10)

$$T=0.5\times U_{1}+0.5\times U_{2}$$ (11)

$$D=\sqrt{C\times T}$$ (12)

Here, *U*_*i*_ represent the EEC and RPH systems, respectively. In the model, *C* denotes the system coupling degree, ranging from 0 to 1, which indicates the match between EEC and RPH. *D* represents the degree of coupling coordination between EEC and RPH, with values also ranging from 0 to 1. Higher values indicate a higher degree of coupling coordination, suggesting a closer and more synergistic relationship between the two systems.

To visually reflect the degree of coupling and coordination between EEC and RPH, this study, inspired by Hou et al. (2022) [42], categorizes the CCD into ten levels, as detailed in Table 2.

**Table 2:** The classification of CCD.

CCD	Classification	CCD	Classification
[0.0, 0.1)	Extreme non-coordination	[0.5, 0.6)	Near coordination
[0.1, 0.2)	Severe non-coordination	[0.6, 0.7)	Primary coordination
[0.2, 0.3)	Moderate non-coordination	[0.7, 0.8)	Intermediate coordination
[0.3, 0.4)	Mild non-coordination	[0.8, 0.9)	Good coordination
[0.4, 0.5)	Near non-coordination	[0.9, 1.0]	Excellent coordination

#### 2.3.3 Dagum Gini coefficient and decomposition method

The decomposition method of the Dagum Gini coefficient, proposed by Dagum in 1997 [43], effectively analyzes the distribution of subgroups and the overlap among sample data, thereby facilitating the decomposition of sources of variation. Initially used to measure income inequality, this method overcomes the limitations associated with traditional Gini coefficients and Theil indices and has been widely applied in the study of economic, environmental, and energy issues [44,45]. This paper employs the Dagum Gini coefficient and its decomposition method to analyze regional disparities in the coupling coordination between EEC and RPH. The specific calculation formulas are as follows:

$$G=\sum_{h=1}^{m}\sum_{i=1}^{m}\sum_{j=1}^{n_{j}}\sum_{k=1}^{n_{i}}\frac{\left|CCD_{hj-}CCD_{ik}\right|}{2n^{2}\delta }$$ (13)

$$G_{w}=\sum_{h=1}^{m}G_{hh}E_{j}s_{j}$$ (14)

$$G_{b}=\sum_{h=2}^{m}\sum_{i=1}^{h=1}G_{hi}D_{hi}(E_{h}s_{i}+E_{i}s_{h})$$ (15)

$$G_{s}=\sum_{h=2}^{m}\sum_{i=1}^{h=1}G_{hm}(1-D_{hi})(E_{h}s_{i}+E_{i}s_{h})$$ (16)

In the model, *m* represents the number of regional subgroups, while *j* and *k* denote the number of provinces within each subgroup. *CCD*_*hj*_ and *CCD*_*ik*_ denotes the CCD between EEC and RPH, with *δ* being the average CCD. The Dagum Gini coefficient decomposes the overall regional disparity G into within-region disparities *G*_*w*_, between-region disparities *G*_*b*_, and transvariation density disparities among regions *G*_*s*_, fulfilling the relationship *G* = *G*_*w*_+*G*_*b*_+*G*_*s*_.

#### 2.3.4 Kernel density estimation (KDE)

In the study of dynamic distributions, KDE is a commonly used non-parametric estimation method. It estimates the probability density function of random variables without assuming a specific form for the underlying data distribution, and is widely used in data visualization and anomaly detection [46]. The kernel density function for random variables, represented as *X*, is given by:

$$f(x)=\frac{1}{nh}\sum_{i=1}^{n}K(\frac{\bar{x}-x_{i}}{h})$$ (17)

Here, *K*(•) represents the kernel function, *X*_1_,*X*_2_,…,*X*_*n*_ denotes the CCD between EEC and RPH for each province in the sample, $\bar{x}$ is the mean, *n* is the number of observations, and *h* is the bandwidth. This paper chooses a Gaussian kernel function, known for its high precision, to estimate the dynamic distribution of the CCD between EEC and RPH across provinces. The function expression is as follows:

$$K(x)=\frac{1}{\sqrt{2\pi }}e^{\left(-\frac{x^{2}}{2}\right)}$$ (18)

#### 2.3.5 Markov chains

After analyzing the overall distribution of the CCD between EEC and RPH, this paper utilizes Markov chains to analyze the dynamic evolution trends of the coordinated development between EEC and RPH in China. Initially, using traditional Markov chain analysis, the internal trend characteristics of the CCD are examined. The Markov transition probability matrix considers transitions between different states as a Markov process. A Markov chain represents a sequence of random variables in a given state space, where the state of the sequence depends only on its current state. The CCD levels are divided into four categories: low, lower-middle, upper-middle, and high, utilizing quartile methods to calculate the transition probability matrix. Then, a Markov transition probability matrix is used to study the evolution laws between different CCD states.

Subsequently, based on spatial correlation analysis, a spatial Markov transition probability matrix (MTPM) is established. The spatial Markov chain incorporates spatial lag conditions into the traditional MTPM. By introducing a spatial weights matrix, the weighted average of neighboring regions is calculated, thereby assessing the spatial lag condition of regional units. This effectively compensates for the traditional Markov chain method’s oversight of mutual influences in neighboring areas [47].

#### 2.3.6 Panel Tobit model

The CCD between EEC and RPH, which ranges from 0 to 1, exhibits truncated and phased characteristics. Using ordinary least squares (OLS) regression could result in biased estimates. The Tobit model is particularly effective in handling truncated data, offering higher estimation precision and reliability. In light of the specific characteristics of the study subject, this paper employs a panel Tobit random effects model to analyze the driving factors of the CCD between EEC and RPH [48].

Drawing from existing research [49–51], factors such as economic development level, urbanization level, degree of openness, education level, and government health expenditure level are selected as external influencing factors. To eliminate dimensional differences and reduce heteroscedasticity and multicollinearity, these variables are logarithmically transformed. The symbols and explanations for each variable are provided in Table 3.

**Table 3:** Influencing factors of CCD.

Variable	Variable Symbol	Variable description	Unit
Economic development level	ln *PGDP*	Per capita GDP	Yuan
Urbanization level	ln *UR*	Urbanization rate of permanent population	%
Degree of openness	ln *OPEN*	Total import and export value of foreign invested enterprises/GDP	%
Education Level	ln *EL*	Number of degrees awarded by general higher education institutions	10^4^ persons
Government health expenditure level	ln *GHE*	Government health expenditure/total social health expenditure	%

## 3 Regional differences and dynamic evolution of CCD

### 3.1 System CCD and regional differences

#### 3.1.1 Measurement and analysis of EEC and RPH levels

**Fig 2:**
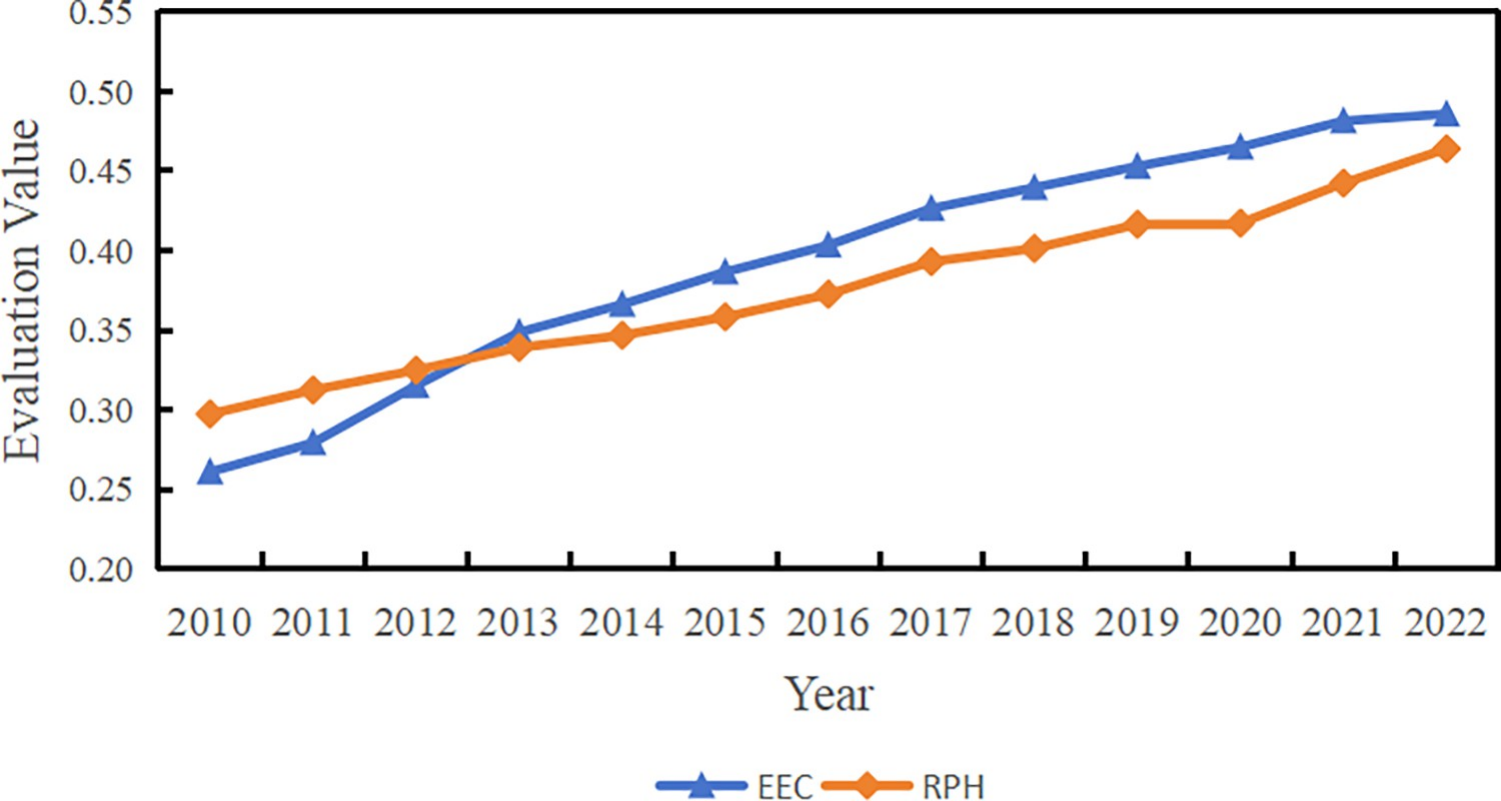
The evaluation value of national EEC and RPH in 2010 to 2022.

This paper employs the entropy method to evaluate the levels of EEC and RPH across 31 provinces in China from 2010 to 2022. The corresponding results are presented in Fig 2.

From the perspective of EEC, during the observation period, EEC showed significant improvement across the country. From 2010 to 2020, the national average level rose from 0.2609 to 0.4858, with a 13-year average of 0.3932, representing an overall growth of 86.20%. Regionally, the Central region exhibited the most notable growth, with an increase of 101.26%, while the Eastern and Western regions experienced growth rates of 80.16% and 83.17%, respectively. Detailed results for the Eastern, Central, and Western regions are presented in Table 4.

**Table 4:** Level of EEC and RPH in the three regions (selected years).

systems	regions	2010	2012	2014	2016	2018	2020	2022	Average
EEC	Eastern	0.2694	0.3272	0.3729	0.4052	0.4406	0.4659	0.4854	0.3981
	Central	0.2342	0.2860	0.3385	0.3799	0.4154	0.4455	0.4713	0.3691
	Western	0.2709	0.3237	0.3784	0.4172	0.4547	0.4772	0.4959	0.4047
RPH	Eastern	0.3314	0.3475	0.3623	0.3909	0.4227	0.4339	0.4922	0.3979
	Central	0.2719	0.3061	0.3210	0.3426	0.3764	0.3954	0.4439	0.3522
	Western	0.2830	0.3167	0.3496	0.3756	0.3984	0.4165	0.4514	0.3713

Comparatively, RPH growth was relatively lower than that of EEC during the same period. Nationally, the level increased from 0.2973 in 2010 to 0.4639 in 2022, with a 13-year average of 0.3758, marking a total increase of 56.05%, 30.15 percentage points lower than the overall growth rate of EEC. Over the 13 years, the growth rate in the Central region was 63.23%, slightly higher than those in the Eastern and Western regions, which recorded growth rates of 48.56% and 59.50%, respectively. For specific levels of RPH in the Eastern, Central, and Western regions, see Table 4.

**Fig 3:**
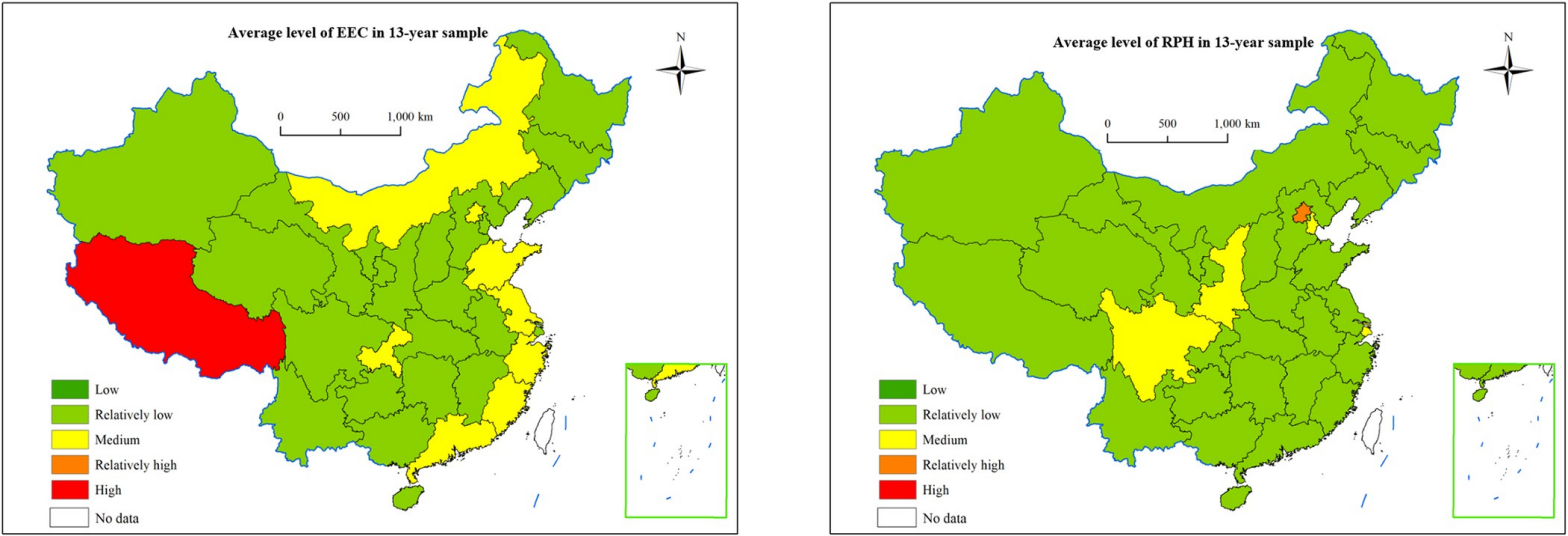
The average level of EEC and RPH in each province. The map is based on the standard map with review number GS (2023) 2767 downloaded from the Standard Map Service website of the Ministry of Natural Resources (http://bzdt.ch.mnr.gov.cn/), with no modifications to the base map.

Looking at the provincial level, the average development levels of EEC and RPH across these provinces over the 13-year sample period are shown in Fig 3.

#### 3.1.2 Results and analysis of the CCD

Based on the modified CCD model, this paper evaluates the CCD between EEC and RPH across 31 provinces in China. Table 5 displays the CCD for the entire country as well as for the Eastern, Central, and Western regions from 2010 to 2022. Nationally, the CCD improved from near non-coordination in 2010 to the grade of primary coordination by 2022. By the end of 2022, the Eastern, Central, and Western regions had all reached a primary level of coordination. However, on average, the CCD during the observation period remained at a near coordination level, both nationally and regionally.

**Table 5:** National and regional results of CCD.

Year	National		Eastern		Central		Western	
	CCD	Classification	CCD	Classification	CCD	Classification	CCD	Classification
2010	0.4908	Near Non	0.5112	Near	0.4733	Near Non	0.4837	Near Non
2011	0.5077	Near	0.5269	Near	0.4895	Near Non	0.5023	Near
2012	0.5365	Near	0.5537	Near	0.5202	Near	0.5316	Near
2013	0.5593	Near	0.5718	Near	0.5485	Near	0.5552	Near
2014	0.5687	Near	0.5719	Near	0.5580	Near	0.5729	Near
2015	0.5802	Near	0.5791	Near	0.5683	Near	0.5891	Near
2016	0.5916	Near	0.5941	Near	0.5759	Near	0.5997	Near
2017	0.6093	Primary	0.6152	Primary	0.5934	Near	0.6145	Primary
2018	0.6143	Primary	0.6167	Primary	0.6029	Primary	0.6196	Primary
2019	0.6272	Primary	0.6243	Primary	0.6194	Primary	0.6350	Primary
2020	0.6301	Primary	0.6357	Primary	0.6207	Primary	0.6312	Primary
2021	0.6466	Primary	0.6532	Primary	0.6406	Primary	0.6446	Primary
2022	0.6595	Primary	0.6667	Primary	0.6578	Primary	0.6541	Primary
Average	0.5863	Near	0.5939	Near	0.5745	Near	0.5872	Near

### 3.2 Spatial differences in CCD and decomposition of origins

Table 6 presents the Dagum Gini coefficient and its decomposition results for the CCD between EEC and RPH. In terms of the overall evolutionary pattern, the CCD exhibits a state of imbalance. However, the overall disparity is decreasing, with the Gini coefficient declining from 0.0316 in 2010 to 0.0199 in 2022, indicating a significant reduction in differences.

**Table 6:** Decomposition of regional differences in CCD.

Year	Total Gini coefficient	Within-region		Between-region		Trans-variation density	
		Origins	Rate	Origins	Rate	Origins	Rate
2010	0.0316	0.0087	27.48%	0.0169	53.49%	0.0060	19.03%
2011	0.0310	0.0086	27.87%	0.0159	51.41%	0.0064	20.72%
2012	0.0242	0.0066	27.48%	0.0135	55.81%	0.0040	16.71%
2013	0.0219	0.0066	30.35%	0.0091	41.50%	0.0062	28.15%
2014	0.0199	0.0063	31.42%	0.0051	25.55%	0.0086	43.02%
2015	0.0234	0.0073	31.22%	0.0077	32.69%	0.0085	36.09%
2016	0.0240	0.0074	30.62%	0.0081	33.77%	0.0086	35.61%
2017	0.0244	0.0075	30.62%	0.0069	28.19%	0.0101	41.19%
2018	0.0256	0.0082	31.95%	0.0054	21.20%	0.0120	46.85%
2019	0.0225	0.0073	32.58%	0.0056	24.69%	0.0096	42.73%
2020	0.0256	0.0085	33.00%	0.0048	18.80%	0.0124	48.20%
2021	0.0216	0.0071	32.69%	0.0042	19.44%	0.0104	47.87%
2022	0.0199	0.0064	32.36%	0.0044	22.23%	0.0090	45.41%
Average	0.0243	0.0074	30.74%	0.0083	32.98%	0.0086	36.28%

Regarding the sources and contributions of disparities, during the observation period, within-region differences have exhibited a relatively stable overall change, with a slight upward trend from 2010 to 2022. The contribution rate of between-region differences has steadily declined, falling from 53.49% in 2010 to 22.23% in 2022. Trans-variation density, which primarily explains the phenomenon of cross-overlap between regions, has generally been on the rise, increasing from 19.03% in 2010 to 45.41% in 2022, contrary to the trend in between-region differences. Consequently, trans-variation density is becoming increasingly significant in explaining the overall change.

### 3.3 Distribution dynamics of CCD

**Fig 4:**
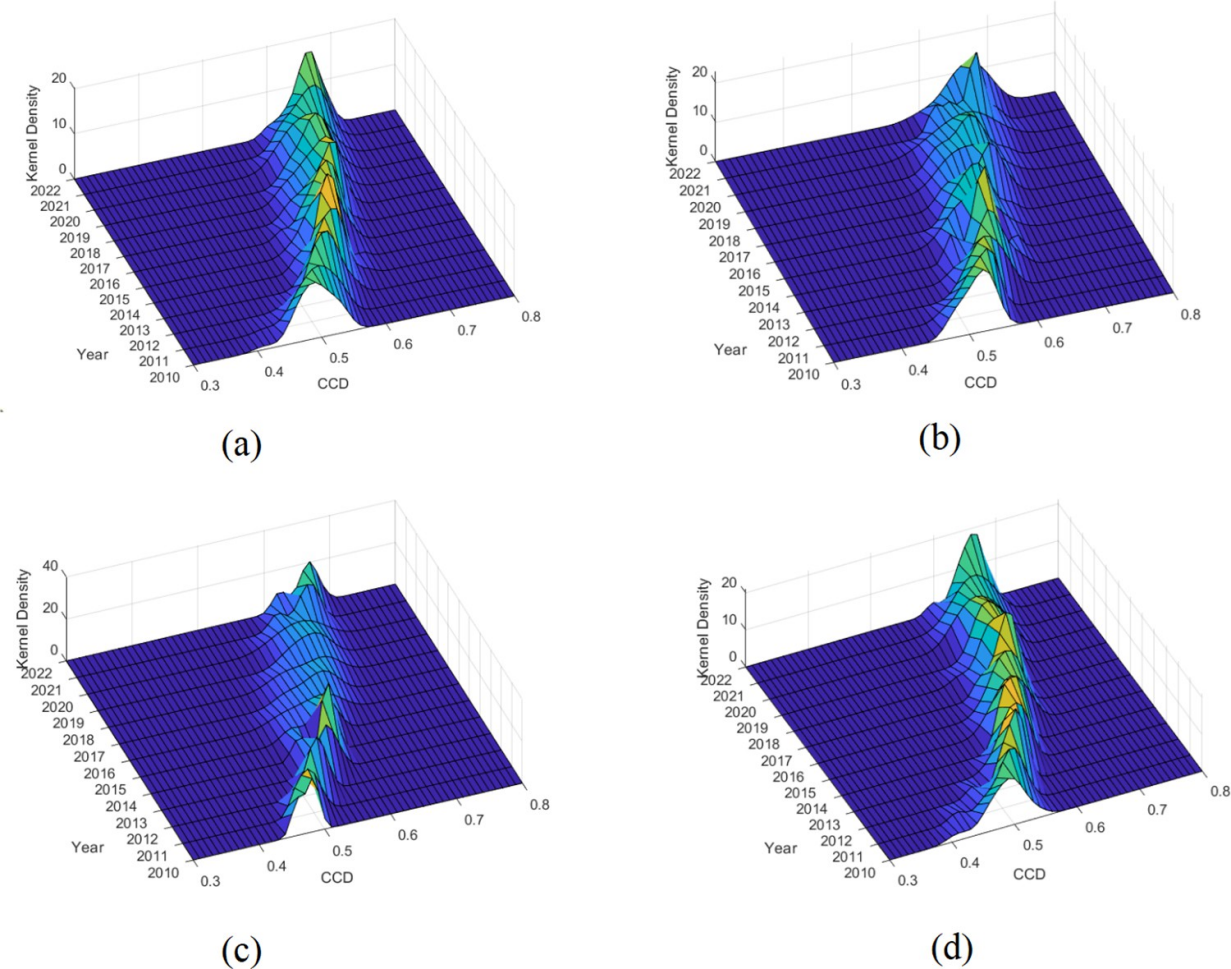
Kernel density estimation of the CCD.

Given the variations in resource endowment and development stages among the Eastern, Central, and Western regions, and across various provinces, the results of CCD at the national level, as well as the trends over time in these three major regions, may differ. Therefore, this study employs KDE (Parzen, 1962) [52] to analyze the dynamic evolution of differences in the coupling and coordination levels between EEC and RPH across the entire sample and the three major regions. This analysis considers three aspects: distribution position, distribution shape, and distribution spread, as illustrated in Fig 4.

Fig 4A displays the distribution dynamics of the CCD between EEC and RPH across the entire sample. It highlights three main characteristics: First, regarding the distribution position, the center of the density curve for the CCD has shifted to the right between 2010 and 2022, indicating a gradual improvement. Second, in terms of distribution shape, the density curve is high and steep, suggesting a trend toward narrowing the absolute differences in CCD levels. Lastly, concerning distribution spread, the right tail of the density curve weakens, indicating a gradual reduction in the variance of CCD among provinces.

Fig 4B–4D describe the dynamic trends of CCD between EEC and RPH in the Eastern, Central, and Western regions during the sample period. First, in terms of distribution position, the evolutionary trends of the three regions are consistent with the overall sample, each showing varying degrees of rightward shifts, suggesting a gradual increase in CCD. Second, regarding distribution shape, the Western region’s kernel density curve exhibits minor fluctuations and little change in width; the Central region’s curve shows a fluctuating downward trend with a noticeable narrowing between 2011 and 2014, followed by a significant increase in spread after 2015. The Eastern region’s overall trend is similar to that of the Central region. Lastly, concerning the number of peaks, the Eastern region presented a bimodal state from 2010 to 2022, indicating significant polarization, likely due to considerable disparities in the levels of coupled coordination development among provinces within the region.

### 3.4 Markov chain analysis of CCD

#### 3.4.1 Traditional Markov chain analysis

**Fig 5:**
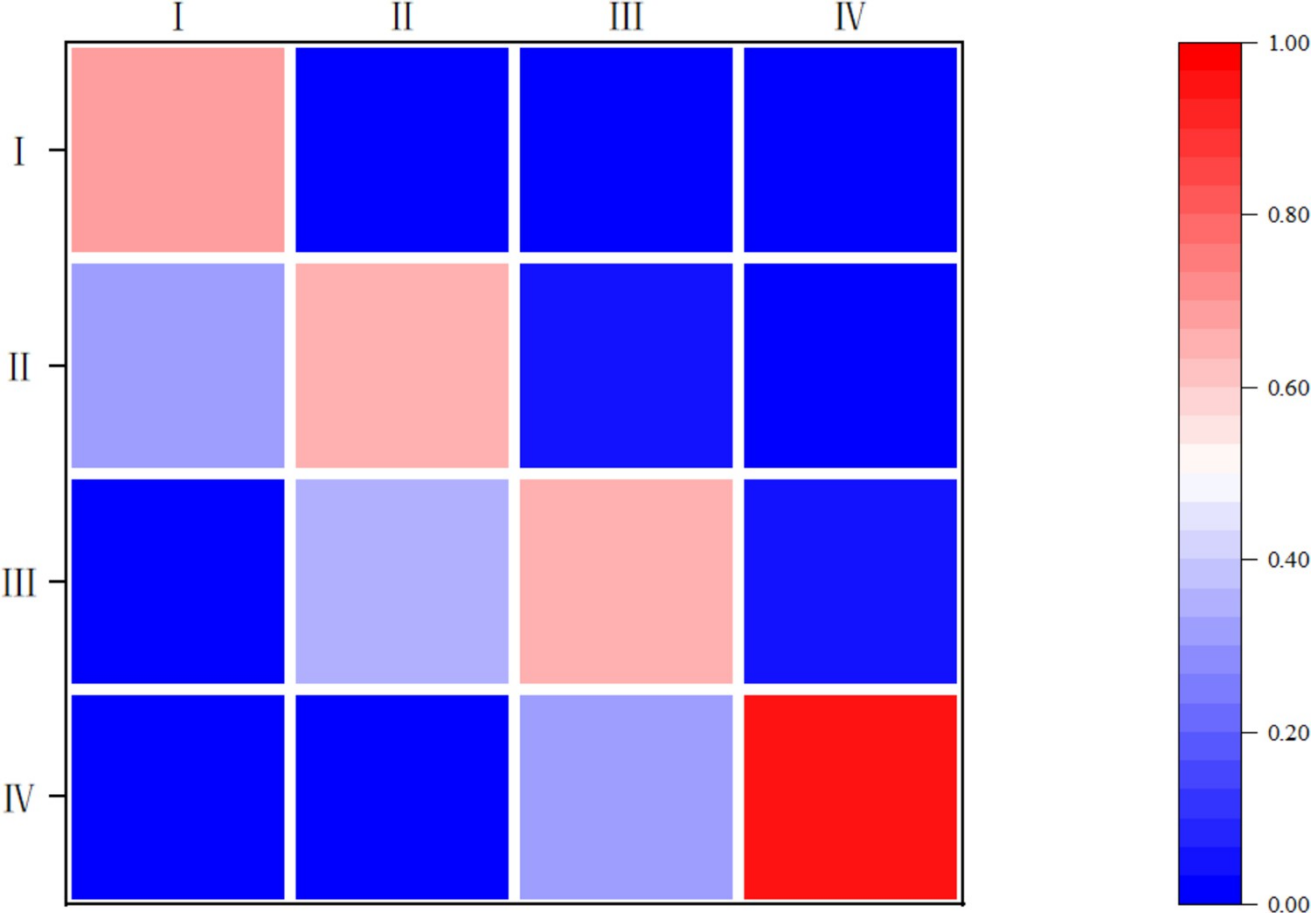
Traditional MTPM for the CCD.

To further examine the internal dynamics and spatial transition characteristics of the CCD between EEC and RPH, this paper introduces the Markov Transition Probability Matrix (MTPM) for analysis. Initially, the traditional Markov chain method is employed to investigate the intrinsic trend characteristics of the CCD between the two systems. The provinces are classified into four distinct levels—low, lower-middle, upper-middle, and high—based on their CCD results, using the quartile method. The resulting transition probability matrix is depicted in Fig 5. Notably, the diagonal elements consistently exceed the off-diagonal elements. Specifically, the probabilities of maintaining the same coupling and coordination level after one year for low, lower-middle, upper-middle, and high levels are 69.31%, 64.36%, 64.58%, and 95.95%, respectively. These results indicate that the CCD levels are relatively stable, leading to a phenomenon of "club convergence". It is also noteworthy that the overall convergence probabilities for the low and high levels are slightly higher compared to the lower-middle and upper-middle levels, suggesting a "Matthew effect" in the CCD between EEC and RPH.

#### 3.4.2 Spatial Markov chain analysis

To explore the spatial relationships in the CCD between EEC and RPH across various provinces, this paper analyzes the CCD data previously calculated. Utilizing Stata 17.0, the global Moran’s I for the CCD over the period 2010 to 2022 was computed, as detailed in Table 7. The results show a significant positive global Moran’s I across the provinces throughout the observation period, suggesting that the level of coupling and coordination in one province is influenced by its neighboring provinces. This influence is characterized by high-high and low-low clustering spatial distribution patterns. Additionally, Moran’s I values were consistently observed between 0.2 and 0.5, demonstrating a stable spatial positive correlation in coupling and coordination.

**Table 7:** Global Moran’s I for CCD (Selected Years).

	2010	2012	2014	2016	2018	2020	2022
Moran’s I	0.315***	0.239***	0.206***	0.375***	0.453***	0.403***	0.248***
Z-value	3.406	2.673	2.367	3.974	4.665	4.178	2.734

Note: ***, **, and * indicating significance at the 1%,5%, and 10% levels, respectively.

**Fig 6:**
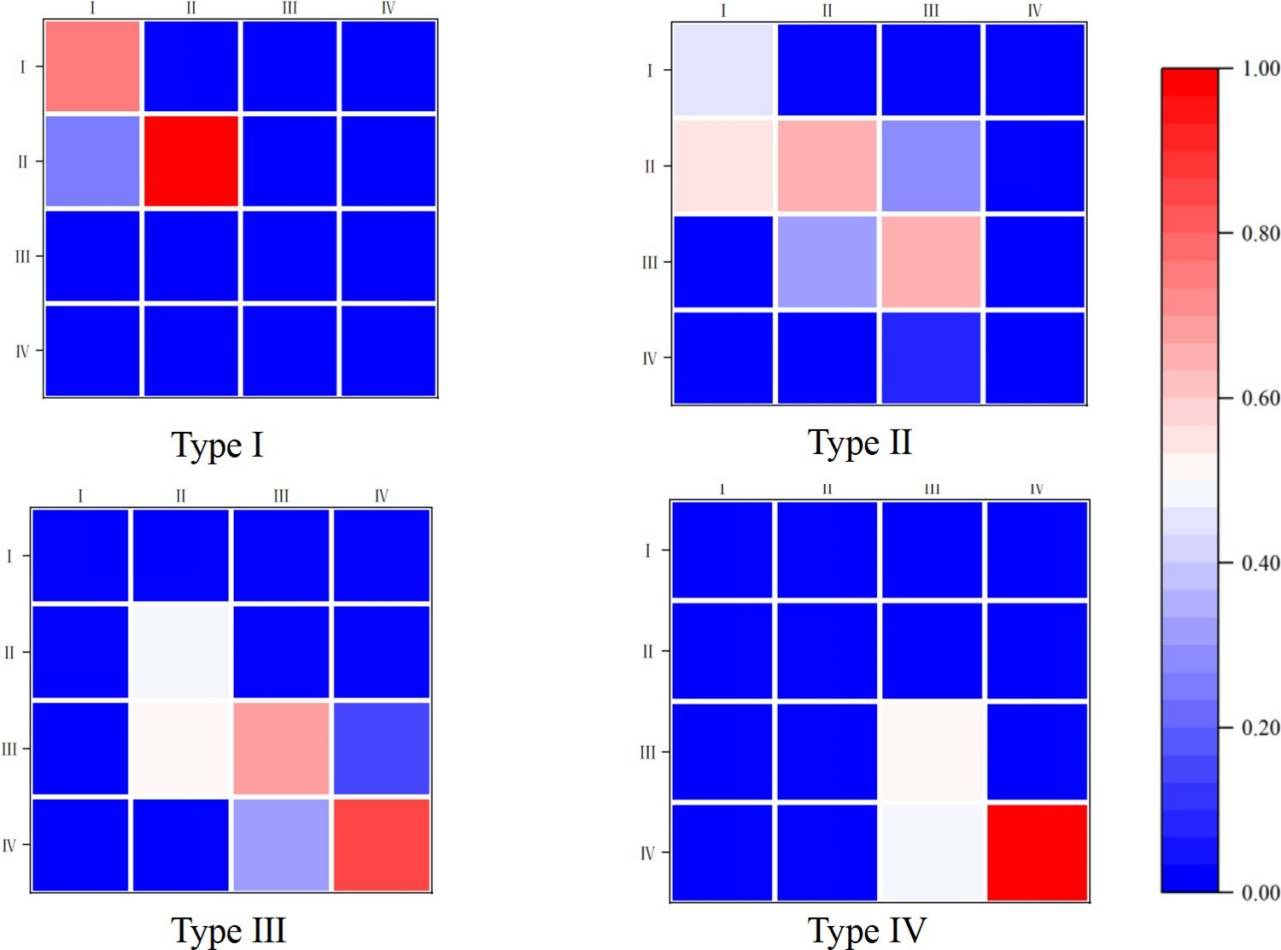
Spatial MTPM for the CCD of EEC and RPH.

These results underline the necessity of incorporating spatial factors and establishing a Spatial MTPM, detailed in Fig 6. First, the four transition probability matrices under different spatial lag types are all distinct, showing that the probability of a province’s coupling and coordination being influenced and transitioning varies in the presence of differences in neighboring provinces. Second, the diagonal elements of the transition matrices under different spatial lag types are not consistently greater than the off-diagonal elements, suggesting that the probability of "status lock" in coupling and coordination decreases under spatial spillover effects. Additionally, non-zero elements exist on both sides of the diagonal, indicating instability in CCD. While upward transitions to an ideal state are possible, there is also a risk of downward transitions, and only adjacent class transitions are feasible, making cross-class jumps difficult. Further, the impact of different lag types on the same level varies. For example, under a low to lower-middle lag type, the probability of transitioning from low to lower-middle is 56.25%, significantly higher than under a low lag type. Lastly, the same lag type impacts different levels differently. Under an upper-middle lag condition, the probabilities of low, lower-middle, and upper-middle levels advancing one level up are 56.25%, 32.43%, and 7.14% respectively, decreasing sequentially, indicating that transition probabilities are influenced not only by the type of lag but also by the initial level of coupling and coordination.

## 4. Analysis of factors influencing CCD

As identified in the previous analysis, notable regional differences exist in the spatial distribution of the CCD between EEC and RPH. Five indicators were selected for examination: economic development level, urbanization level, degree of openness, education level, and government health expenditure level. A panel Tobit model was used to analyze the external factors affecting the CCD. To eliminate the differences in measurement scales and to reduce heteroscedasticity and multicollinearity, logarithmic transformations were applied to these variables. The baseline econometric model is established as follows:

$$\begin{array}{c} CCD_{it}=\alpha +\beta_{1}\;\ln\;PGDP+\beta_{2}\;\ln\;UR+\beta_{3}\;\ln\;OPEN\\ +\beta_{4}\;\ln\;EL+\beta_{5}\;\ln\;GHE+\varepsilon_{it} \end{array}$$ (19)

In the model, *α* represents the constant term, *β*_1_,*β*_2_⋯,*β*_5_ are the coefficients to be estimated, and *ε*_*it*_ is the random error term. The specific meanings of each influencing factor have been introduced previously.

Using Stata 17.0 software, a panel Tobit regression was conducted to examine the factors influencing the CCD between EEC and RPH. The results are presented in Table 8.

**Table 8:** Panel Tobit regression results for factors influencing CCD.

Variables	National	Eastern	Central	Western
ln *PGDP*	0.0845*** (0.0103)	0.0973*** (0.0140)	0.0023 (0.0228)	0.0663*** (0.0177)
ln *UR*	0.145*** (0.0318)	0.0262 (0.0426)	0.362*** (0.0558)	0.209*** (0.0478)
ln *OPEN*	-0.0151*** (0.0025)	-0.0321*** (0.0051)	0.0097 (0.0066)	-0.0121*** (0.0021)
ln *EL*	0.0200*** (0.0073)	0.0013 (0.0081)	0.0694*** (0.0193)	0.0044 (0.0096)
ln *GHE*	-0.0070 (0.0188)	-0.0458* (0.0265)	-0.0005 (0.0304)	-0.0401 (0.0261)
Const	-1.105*** (0.108)	-0.384** (0.194)	-1.727*** (0.213)	-0.843*** (0.146)
Observations	403	143	104	156

Notes:*** p<0.01, ** p<0.05, * p<0.1, Standard errors in parentheses.

Table 8 reveals several key insights into the factors influencing the CCD between EEC and PRH.

1. Nationwide Analysis. All factors, except for government health expenditure level, have coefficients significant at the 1% level. Notably, the degree of openness exhibits a negative coefficient, indicating an adverse correlation with CCD. In contrast, the coefficients for economic development level, urbanization level, and education level are positive, suggesting that these factors contribute positively to the CCD. The ranking of the coefficient magnitudes is as follows: urbanization level > economic development level > education level.
2. Regional Analysis. There is significant regional heterogeneity in the factors influencing CCD across the three major regions. The coefficient for government health expenditure level is negative in all regions, but it is only statistically significant at the 1% level in the Eastern region and not significant in the Central and Western regions. The degree of openness has a significantly negative impact on the Eastern and Western regions, consistent with the nationwide results. However, its impact is positive in the Central region, though not statistically significant. Other indicators, including the levels of economic development, urbanization, and education, all have positive regression coefficients, aligning with the nationwide analysis. This consistency underscore that these three factors positively enhance the CCD between EEC and RPH. However, the specific values and ranking of these factors differ across regions: in the Eastern region, the level of economic development has the most significant positive impact on CCD, while in the Central and Western regions, the urbanization level is the most influential factor, with regression coefficients of 0.362 and 0.209, respectively, both significant at the 1% level.

## 5. Discussion

This study calculated the development levels of EEC and RPH across China’s 31 provinces from 2010 to 2022, including their CCD. Findings reveal that during the study period, both EEC and RPH in China exhibited a stable upward trend, although the overall development level remained relatively low. The CCD showed consistent growth both nationally and across three major regions. Overall inequality in coupling coordination, as measured by Dagum Gini coefficient, has decreased, with the coefficient reducing from 0.0316 in 2010 to 0.0199 in 2022. KDE results indicate a rightward shift in the density curve of the CCD, suggesting a significant reduction in absolute disparities. Panel Tobit regression analysis shows that economic development, urbanization, and education levels significantly and positively influence the CCD on a national scale, with urbanization having the most substantial impact, followed by economic development and education levels.

In the context of global advocacy for green development, sustainable development, and enhancement of overall health standards, the conclusions of this paper provide valuable insights into the coupling and coordination levels and dynamic trends of China’s EEC and RPH. Previous research [42] has also explored the coupling and coordination relationship between China’s ecological environment and health systems, revealing that ecological and health systems often reinforce each other. This is consistent with the theme of this study. This research contributes important theoretical foundations and policy recommendations for promoting the coupled and coordinated development of EEC and RPH, both in the present and in the future. The findings of this research warrant further in-depth discussion to explore more nuanced implications and practical applications. By doing so, policymakers can better integrate sustainable environmental practices with public health improvement efforts, thus facilitating more balanced and long-term development in both domains.

First, throughout the observation period, both EEC and RPH showed continuous improvement. Specifically, EEC grew by 86.20%, while RPH increased by 56.05%. Numerous existing studies support the findings of this paper, indicating a sustained improvement in the quality of China’s ecological environment [53,54]. The growth in EEC can largely be attributed to the government’s increased focus on pollution prevention and control, alongside a broader societal understanding of the importance of green development. The Chinese government has prioritized addressing environmental pollution, enacting various policy documents aimed at managing pollution violations, and investing heavily in ecological restoration efforts. Furthermore, to expedite carbon reduction, the government has set dual carbon goals, which have also positively impacted the enhancement of ecological civilization. At the same time, RPH levels also improved, though at a slower rate than EEC. The RPH index system constructed in this paper includes dimensions such as medical personnel, medical facilities, healing capacity, and medical efficiency, covering various aspects. Although the government places great importance on improving public health, and invests significantly each year to enhance the medical environment, the large population base in China means that RPH development is relatively slow on average.

Second, from a temporal perspective, the CCD between EEC and RPH showed a stable increase. By 2022, the CCD levels nationally and across the three major regions had all reached a primary level of coordination. Comparatively, the Eastern region had a slightly higher level of CCD than the Central and Western regions. This disparity is understandable, as the Eastern region, consisting mostly of coastal provinces, has abundant technological, capital, and health resources and a higher level of social development. While rapidly developing economically, this region also focuses on improving EEC and RPH, exhibiting high coordination.

Third, this study analyzes the internal dynamics and spatial transition characteristics of the CCD between EEC and RPH using both traditional and spatial MTPM approaches. The traditional MTPM results indicate that the CCD is relatively stable and exhibits a "club convergence" phenomenon. Meanwhile, the spatial MTPM reveals that a province’s CCD level is influenced by the CCD levels of its neighboring provinces. Under the influence of spatial spillover effects, the likelihood of a "status lock" in the CCD is reduced. Through this dynamic Markov chain analysis, this paper uncovers both the stability and fluctuation characteristics of the CCD between EEC and RPH across Chinese provinces, further highlighting the influence of spatial factors on the CCD’s evolutionary trajectory. Notably, by strengthening inter-regional linkages, overall improvements in coupling and coordination can be achieved more effectively, particularly in regions with lower levels of economic development, where targeted policy support and regional cooperation are essential for fostering progress. Additionally, the findings of this study contribute valuable insights to other research on spatial spillover effects [55], which also demonstrate that neighboring regions significantly influence each other’s development through spatial interactions. This further emphasizes the role of spatial dependence in shaping regional development patterns.

Fourth, factors influencing the CCD between EEC and RPH have been examined in this study. Panel Tobit regression results at the national level reveal that the influence of government health expenditure level, represented as the ratio of government health spending to total health expenditure, on CCD is not significant. A possible explanation for this is that the proportion of government health spending within the total health expenditure is relatively low. Currently, social health expenditure accounts for the highest proportion of total health expenses, and the government is continually increasing its health investments to reduce the personal health expenditure burden. Additionally, at the national level and in both the Eastern and Western regions, the coefficient for the degree of openness is negative. One possible interpretation is that while an increased level of openness can effectively promote regional economic growth through investment in capital and talent, and potentially enhance local public health through the introduction of medical technology, its direct effect on pollution control and ecological environment improvement is relatively minor. Therefore, it has not effectively promoted the enhancement of coupling and coordination between EEC and RPH. Furthermore, other research supports the findings of this study’s Tobit regression. For instance, one study [56] focused on the relationship between urbanization and public health efficiency discusses the role of urbanization, economic development, and other factors in promoting health improvements, which resonates with the results of this study’s Tobit model.

Besides discussing the research content, this paper also offers significant new contributions. Firstly, this paper adopts an innovative research approach by presenting a new quantitative analysis perspective. This is the first time a modified CCD model has been used to analyze the dynamic relationship between EEC and RPH, aiding relevant departments in better understanding the coordinated development between these areas. Secondly, the paper further analyzes the spatial patterns of CCD between EEC and RPH and their influencing factors. The Tobit regression results provide a theoretical basis for the Chinese government to formulate policies and measures to promote coordinated development between EEC and RPH.

## 6. Conclusions and future research directions

### 6.1 Conclusions

This study investigates the coupling coordination between EEC and RPH across 31 provinces in China from 2010 to 2022. Using a modified CCD model, alongside tools like the Dagum Gini coefficient, KDE, Markov chains, and panel Tobit regression, the study offers insights into their dynamic relationship and influencing factors.

Firstly, both EEC and RPH showed a steady upward trend during the study period, with EEC growing by 86.20% and RPH by 56.05%. Despite these improvements, overall development levels remain relatively low, suggesting room for further enhancement. Secondly, a consistent rise in CCD was observed nationally and across the three major regions, progressing from near non-coordination in 2010 to primary coordination by 2022. The regional disparities, as indicated by the Gini coefficient, narrowed over time, primarily driven by reduced inter-regional differences. Thirdly, KDE results revealed a rightward shift in the density curve, reflecting overall improvements in CC, while spatial autocorrelation tests confirmed stable positive spatial correlations with high-high and low-low clustering patterns. Fourthly, traditional MTPM results indicate that the CCD is relatively stable and exhibits a "club convergence" phenomenon. Meanwhile, spatial MTPM reveals that a province’s CCD level is influenced by the CCD levels of its neighboring provinces. Under the influence of spatial spillover effects, the likelihood of a "status lock" in the CCD is reduced. Finally, the panel Tobit regression showed that urbanization, economic development, and education positively influenced CCD, with urbanization having the strongest effect. The degree of openness negatively impacted the CCD, and government health expenditure had no significant effect.

### 6.2 Policy implications

Firstly, strengthening environmental regulations is crucial. Given the positive correlation between EEC development and public health, stricter control on industrial emissions and wider adoption of pollution control technologies should be prioritized. This will help address the environmental challenges highlighted in the study and foster better public health outcomes.

Secondly, increasing investment in healthcare infrastructure is vital, particularly in underserved regions. Improved healthcare facilities and public health campaigns promoting preventive healthcare can enhance public health. Simultaneously, it is essential to manage the adverse effects of globalization. While globalization can bring technological and economic benefits, it may also exacerbate pollution. Stricter environmental regulations can mitigate these risks, ensuring that economic growth contributes positively to both the environment and public health.

Finally, tailored regional approaches are needed to address local disparities. Policies should be region-specific, considering economic and environmental conditions unique to each area. Continuous monitoring and evaluation will be essential for adapting these policies to evolving circumstances, ensuring alignment with both immediate needs and long-term sustainable development goals.

### 6.3 Limitations and outlook

Despite the contributions mentioned earlier, this research also has certain limitations. First, although the most recent available data was used, data for 2023 could not be obtained, which limited the study’s ability to analyze and discuss the latest trends from that year. Second, while this study focused on the 31 provinces of China, exploring the CCD between EEC and RPH at more granular levels, such as at the city or county level, holds considerable practical significance and value. However, this aspect remains a gap in the current research and represents limitations of this study. Naturally, this also suggests an important direction for future research to expand upon.

## Supporting information

S1 Dataset (XLSX)
